# Prevalence, Forms, and Factors Associated With the Use of Herbal Medicine Among Adults Diagnosed With Hypertension at Naguru Regional Referral Hospital: An Analytical Cross‐Sectional Study

**DOI:** 10.1155/jotm/2510086

**Published:** 2025-12-19

**Authors:** Keneth Mugume, Florence Nakaggwa, John Bosco Alege, Rose Clarke Nanyonga

**Affiliations:** ^1^ School of Public Health, Clarke International University, P.O. Box 7782, Kampala, Uganda, ciu.ac.ug

## Abstract

**Background:**

Previous studies reported increasing herbal medicine (HM) use among patients with cardiovascular diseases, including hypertension. Although HMs are widely used for their therapeutic benefits, repeated use has raised concerns due to reported adverse effects and potential interactions with conventional treatments. This study assessed the prevalence, forms (preparation), and factors associated with HM use among hypertension patients in an urban setting in Uganda.

**Methods:**

An analytical cross‐sectional study was conducted in March 2021 among 121 patients with hypertension at Naguru Regional Referral Hospital using standardized questionnaires. Descriptive and multivariate analyses identified factors independently associated with HM use, with *p*  <  0.05 as the significance threshold.

**Results:**

All 121 (100%) participants had ever used HM for hypertension; 86 (71.1%) reported consistent use in the past 12 months. Daily use was reported at 53 (43.8%), while 74 (61.2%) reported concurrent use with conventional medicine. The majority used HM in the liquid form [74 (61.2%)], but most HMs were ingested orally [101 (83.5%)]. 53 participants (43.8%) chose HMs themselves, and 46 (39.3%) procured from herbalists. The main HM knowledge source was family [53 (25.6%)]. High odds of HM use were observed among participants from the Central Region (AOR = 9.4; 95% CI = 1.7–51.0), those with easy access to herbalists and doctors (AOR = 1.6; 95% CI = 0.4–76.8), those who did not inform health workers about HM use (AOR = 5.3; 95% CI = 0.5–54.0), and those who perceived concurrent HM use as safe and beneficial (AOR = 2.2; 95% CI = 0.6–7.3) or free of side effects (AOR = 6.8; 95% CI = 1.6–28.9). Region (*p* = 0.009), accessibility (*p* = 0.049), and perception of no side effects (*p* = 0.009) were significantly associated with HM use.

**Conclusion:**

HM is widely used by urban‐dwelling patients with hypertension and is influenced by perceived benefits, safety, and ease of access. Health workers need to proactively inquire about HM use to effectively advise hypertension patients, tailor interventions appropriately, and monitor treatment outcomes. More research is needed to systematically measure and track outcomes in patients who use HM remedies to treat hypertension.

## 1. Introduction

Cardiovascular diseases (CVDs) are among the leading causes of morbidity and mortality among adults globally, with hypertension ranking as the highest [1]. In sub‐Saharan Africa, about 80% of the deaths from CVDs occur among low‐ and middle‐income earners [2]. Hypertension, in particular, is an important contributor to the global burden of disease and mortality and is a growing public health problem in sub‐Saharan Africa [3]. In Uganda, Guwatudde et al. [4] reported the overall prevalence of hypertension at 26.4%. Prevalence among urban versus rural dwellers was reported at 28.9% and 25.8%, respectively. A follow‐up study [5] reported the age‐ and sex‐adjusted prevalence of hypertension at 31.5% (95% confidence interval [CI] 30.2–32.8), with the highest prevalence in the Central Region (34.3%; 95% CI 32.6–36.0).

Treatment and control of hypertension remain suboptimal in most sub‐Saharan African countries due to factors such as limited healthcare infrastructure, poor access to medications, and clinical inertia [6, 7]. Control rates are often below 25%, contributing to a high CVD burden. Barriers, including inconsistent screening and sociocultural challenges, further hinder management, and strategies such as task shifting and community interventions are essential to improve outcomes [6, 7]. This is largely due to budget and capacity constraints across the health systems [8]. Patients increasingly use herbal medicine (HM), a complementary and alternative medicine (CAM), as an alternative intervention to meet their therapeutic needs [9, 10]. In their systematic review, researchers [11] reported three top reasons for CAM use, including expecting benefits, dissatisfaction with conventional medicine, and the perceived safety of CAM. References [12, 13] cited indigenous knowledge of both medicinal plants and traditional cures, leading to widespread acceptance and use of HM. Other reasons for these choices are not fully understood and vary from country to country [14]. However, Refs. [15, 16] indicated that the use of HM products and supplements has increased over the past 3 decades, with more than 80% of patients worldwide relying on them for some or all of their primary health care. Up to 80% of the population in Africa has been reported to use HM to meet their healthcare needs [17].

Access to herbalists with specialized knowledge of HM is a documented key enabler. In Uganda, for example, the ratio of herbal practitioners to population is between 1:200 and 1:400 [18]; in contrast, the ratio of conventional practitioners to population is 1:20,000 or less [18]. As a result, herbal practitioners are readily accessible and often well accepted within the community. The use of herbs is also highly popular and firmly embedded in the belief systems of many societies, and communities utilize herbal therapies as first‐line interventions for many ailments [19]. Studies [14, 20] evaluating hypertension treatment in Uganda have reported that a significant number of persons with hypertension use alternative therapies and that the use of HM is a factor that influences health‐seeking behaviors for hypertension care.

The World Health Organization (WHO) defines traditional medicine as health practices, approaches, knowledge, and beliefs that incorporate plant, animal, and mineral‐based medicines, spiritual therapies, manual techniques, and exercises, applied singularly or in combination to treat, diagnose, and prevent illnesses and maintain well‐being [18]. The use of HM poses challenges for primary healthcare providers, including the fact that the standards of traditional herbal practitioners are currently unknown [21]. Existing guidelines by the National Drug Authority (NDA) [22] are limited to clinical research on HM products and are not necessarily for end‐user consumption. Patients’ preference for HM has also been reported as an increasing barrier to medication adherence [23]. Importantly, Ref. [10] reported that most people who use alternative therapies do not always disclose usage to their primary healthcare providers due to the fear of or negative attitudes from healthcare providers. Related challenges for providers and HM users include concerns about potential benefits and adverse effects. Some studies [15, 24, 25] highlighted that herbal remedies can cause adverse effects ranging from serious injuries to life‐threatening conditions and even death, warranting careful checks and open discussions about HM among patients and providers.

The use of HM in Uganda among hypertension patients is minimally documented. A recent study of hypertension patients in the rural districts of Buikwe and Mukono found that about 14.3% of participants were using HM in combination with conventional medicine [14]. However, the reasons for the adoption of herbal therapies by adult patients with hypertension in an urban setting are not well documented. Uganda is experiencing rapid urbanization, with an annual urban growth rate of about 5.2%, resulting in 27% of the population living in cities as of 2023 and an anticipated addition of over 8 million people to urban areas by 2030 [26]. This swift growth emphasizes the changing dynamics of urban environments, which differ from rural areas in terms of healthcare infrastructure, socioeconomic factors, and disease patterns. Urban centers face specific health challenges, including a higher prevalence of noncommunicable diseases (NCDs) such as hypertension, driven by lifestyle changes and environmental factors linked to city living [27]. Moreover, many urban residents live in informal settlements with limited access to essential healthcare, resulting in disparities in health outcomes [28]. These factors highlight the need for research explicitly focused on urban populations to understand unique health behaviors and treatment choices, including access to, use of, and disclosure practices related to HM, with implications for hypertension care, outcomes, and targeted public health strategies.

The purpose of this study was to assess the prevalence, forms (presentations), and factors linked to HM use among adults with hypertension attending the outpatient clinic at Naguru General Hospital in Uganda. Such data are crucial to inform policymakers’ growing interest in establishing parallel HM standards and safety monitoring practices for patients and clinicians.

## 2. Materials and Methods

This analytical cross‐sectional study was conducted among adult (≥ 18 years) patients attending the hypertension outpatient clinic at Naguru General Hospital (also known as China‐Uganda Friendship Hospital, Naguru).

### 2.1. Study Setting

Naguru General Hospital, also known as China‐Uganda Friendship Hospital, Naguru (CUFH‐N), is located in Nakawa Division, which is the largest of the five divisions in Kampala District, Uganda’s Capital. Naguru Referral Hospital is jointly governed by the Ministry of Health and Kampala Capital City Authority (KCCA) and primarily serves the residents of Nakawa Division, Kampala Metropolitan Area, and other patients referred to the facility. According to the 2014 constituency profiles, Nakawa Division had 317,023 people out of a total of 1,507,080 in Kampala. The hospital provides inpatient, outpatient, diagnostic, prevention, rehabilitation, community outreach, management and support, and specialized curative care services, including urology, gastroenterology, orthopedics, acupuncture, ENT, diabetes, hypertension, and specialized pediatrics. The hospital offers a 24‐h service to all clients. The target population for the study was adult clients diagnosed with hypertension attending the hypertension outpatient clinic.

Naguru’s early establishment, its specific catchment area, and its role in receiving referrals from KCCA Health Centers (IV, III, and II) as well as in easing congestion at Mulago Hospital [29] made it a key location for studying urban healthcare use, including HM consumption.

### 2.2. Inclusion Criteria

The following criteria were used to determine the eligibility:

I. patients diagnosed with hypertension and
II. patients aged 18 years and above.

Respondents with a known history of impaired mental capacity to provide coherent and reliable information were excluded from participating in the study. Participants who declined consent at any stage of the screening process were also excluded from the study.

### 2.3. Sample Size Estimation

The sample size was determined using Cochran’s formula for estimating a single population proportion at a 95% confidence level. An expected proportion of 33% was assumed, reflecting prior evidence on the prevalence of HM use among patients with a chronic illness [30]. A precision level of 9% was applied, and the corresponding confidence interval was (*Z* = 1.96). This yielded an initial sample size of approximately 103 participants. After adjusting for a 10% nonresponse rate, the final required sample size was 120 participants. A 9% precision level was selected to ensure a feasible sample size, given the available resources, while still allowing for a reasonably accurate estimate of the outcome of interest.

### 2.4. Sampling

On arrival at the outpatient hypertension clinic, the patients’ names, which were usually recorded in the patient log, were selected using systematic random sampling; these patients were then approached by the research assistants for recruitment into the study. Systematic random sampling ensured that all available clients had an equal probability of enrollment into the study. To determine the sampling interval, the average number of hypertension patients seen weekly was divided by the required weekly number of participants to obtain the interval. On average, 363 patients were listed in the clinic log. We contacted every third patient on this list, yielding a sample of 121 participants. For those who declined to participate, we moved on to the next eligible participant. However, we did not encounter any refusals.

### 2.5. Data Collection

A structured written research questionnaire was used by two trained research assistants to collect data on the established diagnosis of hypertension, factors associated with HM use, a brief profile (forms) of HMs used by the participant, and any perceived effects (benefits or side effects) of using HM. The questions were modified from the International Questionnaire on the Use of Complementary and Alternative Medicine (I‐CAM‐Q), Section 3 (The use of Herbal Medicine and Dietary Supplements) [31], which has been adapted and validated [32, 33] for use across different countries. The research questionnaire was subsequently pretested on volunteer hypertension patients attending an outpatient clinic in Naguru Hospital before the actual data collection, and necessary adjustments were made.

Data were collected on sociodemographic factors, including age, sex, education level, and employment. Additional data were collected on the source of knowledge about herbs; source of herbs used; forms of herbal preparations, e.g., solutions, ointments, oils, and powders; frequency of use; and mode of administration. Other factors contributing to the use of HM were assessed (the cost of conventional medicine, access to HM, the perception that it is safe and beneficial, perceptions about side effects, and communication with health workers about HM use). In terms of prevalence, we assessed all time use (participants have used HM to treat hypertension at least once); the HM used daily, weekly, and in the past 12 months (either used with conventional treatment or used instead of conventional treatment of hypertension).

### 2.6. Statistical Analysis

Data were captured and entered into a Microsoft Excel sheet for ordering and cleaning and then exported for analysis in SPSS (Version 26).

Univariate analysis was used to summarize all categorical variables as frequencies and percentages. Using a bivariate analysis, the researchers tested for associations between the use of HM and (a) enabling factors (employment, cost of both conventional and HM, access, and communication with health workers) and (b) predisposing factors (age, gender, education level, employment status, perceptions of safety, and perceptions of side effects). In the multivariate analysis, variables that were significantly associated (*p* value 0.05) at the bivariate analysis stage were included as potential confounders in the multivariate regression model to determine the factors independently associated with the use of HM. A *p* value of < 0.05 was considered statistically significant.

### 2.7. Ethical Considerations

When a potential participant expressed interest in participating in the study, informed consent was obtained. Approval to conduct this study was secured from the Research and Ethics Committee of Clarke International University (Number: CLARKE‐2021‐70). Institutional approval was also sought from the Research and Ethics Committee at Naguru Regional Referral Hospital. Before being recruited into the study, the research assistant provided clear explanations of the study’s nature, purpose, and possible benefits and risks to potential participants. Following this, voluntary written consent was collected. A copy of the signed consent form was provided to each participant.

## 3. Results

The demographic characteristics of the 121 participants are given in Table 1. Overall, 80 (66.1%) participants were female, 46 (38.0%) were above 55 years of age, and 49 (40.5%) had primary education as their highest level of education. The majority of the participants [67 (55.4%)] were employed. Although all participants lived in the urban center, 49 (40.5%) identified the Central Region as their place of origin. Catholic participants, [64 (52.9%)] reported the highest use of HM (Table 1).

**Table 1:** Social demographic characteristics of the study participants, *N* = 121.

Variable		Frequency (no. of participants)	%
Age	18–24	2	1.7
	25–34	13	10.7
	35–44	28	23.1
	45–54	32	26.5
	≥ 55	46	38.0

Sex	Male	41	33.9
	Female	80	66.1

Education status	Never attended	16	13.2
	Primary	49	40.5
	Secondary	47	38.8
	Tertiary	9	7.4

Employment status	Employed	67	55.4
	Not employed	54	44.6

Residence	Urban	121	100
	Rural	0	0.0

Region of origin	Northern	15	12.4
	Eastern	25	20.7
	Central	49	40.5
	Western	32	26.4

Religion	Anglican	23	19.0
	Pentecostal	17	14.1
	Catholic	64	52.9
	Muslim	12	9.9
	Other	5	4.1

### 3.1. Prevalence of HM Use

In this study, all 121 (100%) participants had ever used HM. Of these, 86 (71%) reported using HM for hypertension treatment in the last 12 months, while 35 (28.9%) had used HM at least once in their lifetime but not in the past 12 months. The frequency was further divided into daily, weekly, and monthly schedules. At least 53 (43.8%) participants used HM daily; 74 (61.2%) reported using HM in combination with conventional medicine for hypertension treatment; 31 (25.6%) used conventional medicine alone for hypertension treatment, while 12 (9.9%) used HM alone for hypertension treatment.

### 3.2. Preparations of HM

Liquid (mixtures) and oils were the most common 74 (62%) forms (preparations) of HM used, and 101 (83.5%) reported oral ingestion as the highest mode of administration. 53 (44%) of the participants reported having used HM daily for the treatment of hypertension. The main sources of knowledge about HM were family [53 (25.6%)], friends [48 (23.2%)], herbalists [48 (23.2%)], and media [41 (19.8%)]. A moderate [53 (44%)] of participants reported picking HM on their own (Table 2).

**Table 2:** Preparations and presentation, administration modes, and sources of herbal medicine used among study participants (*N* = 121; multiple responses allowed for source‐related items).

Variable	Characteristic	No. of participants	Percentage
Forms (preparations) of herbs used	Liquid form, e.g., liquid mixtures and oils	74	61.2
	Solid form, e.g., Clay, seeds, roots, leaves	32	26,4
	Smoke from, e.g., steam, aroma	3	2.5
	Pastes	9	7.4
	Mixed formulation	3	2.5

Mode of administration	Oral ingestion	101	83.5
	Body smearing/bathing	2	1.7
	Other	18	14.9

Source of knowledge about herbal medicine^∗∗^	Family member	53	25.6
	Community elder	4	2.0
	Herbalist	48	23.2
	Friend	48	23.2
	Advert media	41	19.8
	Other	13	6.2

Source of herbal medicine^∗∗^	Picked it yourself	53	44
	From a friend	20	17.1
	From a herbalist	46	39.3
	From a herbal medicine retailer	14	12.0
	Other	17	14.5

^∗∗^Percentages are based on total responses rather than total participants; thus, they may exceed 100%.

In the bivariate analysis (Table 3), participants aged 35–44 years reported the highest use of HM 20(75%) in the last 12 months. Participants with secondary school education used HM more frequently, and the overall level of education was significantly associated with HM use (*p* = 0.031). Although the majority of unemployed participants reported having used HM in the last 12 months, employment status was not associated (*p* = 0.803) with HM use. Participants from Central Uganda, 49 (40.5%), and those who were Catholics, 64 (52.9%), constituted the highest proportion of HM users compared to participants from other regions and religions, respectively; region of origin was statistically associated with HM use (*p* = 0.046).

**Table 3:** Factors associated with the use of herbal medicine among study participants.

Factors		Total, *n* = 121	Herbal medicine use in the last 12 months		*p* value
			No, *n* (%)	Yes, *n* (%)	
			35 (28.9)	86 (71.1)	

Age categories	18–24	2	2 (100)	0	0.358
	25–34	13	4 (30.8)	9 (69.2)	
	35–44	28	7 (25.0)	21 (75.0)	
	45–54	32	9 (28.1)	23 (71.9)	
	≥ 55	46	13 (28.3)	33 (71.7)	

Gender	Male	41	12 (29.3)	29 (70.7)	0.953
	Female	80	23 (28.7)	57 (71.3)	

Level of education	Never attended	16	3 (18.7)	13 (81.3)	0.031^∗^
	Primary	49	20 (40.8)	29 (59.2)	
	Secondary	47	8 (17.0)	39 (83.0)	
	Tertiary	9	4 (44.4)	5 (55.6)	

Employment	Yes	67	20 (29.8)	47 (70.2)	0.803
	No	54	15 (27.8)	39 (72.2)	

Region of origin	Northern	15	7 (46.7)	8 (53.3)	0.046^∗^
	Eastern	25	10 (40.0)	15 (60.0)	
	Central	49	8 (16.3)	41 (83.7)	
	Western	32	10 (31.2)	22 (68.8)	

Religion	Anglican	23	11 (47.8)	12 (52.2)	0.274
	Pentecostal	17	5 (29.4)	12 (70.6)	
	Catholic	64	15 (23.4)	49 (76.6)	
	Muslim	12	3 (25.0)	9 (75.0)	
	Other	5	1 (20.0)	4 (80.0)	

Cost of medicine	Herbs are expensive	32	9 (28.1)	23 (71.9)	0.907
	Conventional drugs are expensive	89	26 (29.2)	63 (70.8)	

Accessibility	Herbalists are more accessible	62	11 (17.7)	51 (82.3)	< 0.001^∗^
	Doctors are more accessible	32	18 (56.2)	14 (43.8)	
	Both	27	6 (22.2)	21 (77.8)	

The perception that it is safe and beneficial to use herbal medicine and conventional medicine together	No	29	16 (55.2)	13 (44.8)	< 0.001^∗^
	Yes	92	19 (20.6)	73 (79.4)	

Perception of the absence of side effects of herbs	No	87	32 (36.8)	55 (63.2)	0.002^∗^
	Yes	34	3 (8.8)	31 (91.2)	

Communication with health workers about herbal medicine use	Yes	8	4 (50.0)	4 (50.0)	0.096
	No	91	28 (30.8)	63 (69.2)	
	Do not remember	22	3 (13.6)	19 (86.4)	

^∗^Significant at *p* value < 0.05.

The majority of participants reported that herbalists [62 (51.2%)] were more accessible compared to conventional health workers [32 (19.0%)], and access was significantly (*p* = 0.001) associated with HM use. Although the majority of participants reported that conventional medicine for hypertension was costlier, 89 (73.6%), compared to HM, cost/affordability was not significantly (*p* = 0.907) associated with the use of HM. Of those using HM in the last 12 months, the majority had a positive perception of the concurrent use of both herbal and conventional medicine in the treatment of hypertension [73 (79.4%)]. Relatedly, the perception that it is safe and beneficial to use both concurrently was significantly associated with HM use (*p* = 0.001). Regarding side effects, participants were asked about their perceptions of those associated with HM use. The majority of participants reported no side effects from HM use (or concurrent use), and their perception of the absence of side effects was significantly associated with HM use (*p* = 0.002). Finally, the majority of participants reported not notifying/communicating with conventional health workers about HM use; however, this was not significantly associated with HM use (*p* = 0.096) (Table 3).

### 3.3. Multivariate Analysis

A multivariate logistic regression analysis was conducted with 95% CIs to identify factors associated with HM use. Variables with significant associations (*p* < 0.05) in preliminary analyses were considered for inclusion in the multivariate model. The odds of HM use in the last 12 months were higher among participants from the Central Region (AOR = 9.4; 95% CI = 1.7–51.0), those who reported that both herbalists and doctors were equally accessible (AOR = 1.6; 95% CI = 0.4–76.8), participants who perceived that using both conventional and HM concurrently is safe and beneficial (AOR = 2.2; 95% CI = 0.6–7.3), participants who perceived the absence of side effects from the use of HM (AOR = 6.8; 95% CI = 1.6–28.9), and those who did not notify/communicate with conventional health workers about the use of HM (AOR = 5.3: 95% CI = 0.5–54.0). After adjusting for the education level, region of origin, accessibility, perception of safety, benefits and side effects, and communication with health workers about HM use, coming from the Central Region (*p* = 0.009) compared to the Northern Region, accessibility to doctors (*p* = 0.049) compared to accessibility to herbalists, a perception of the absence of side effects from the use of HM (*p* = 0.009), and failing to remember whether the participant notified/communicated with conventional health workers about the use of HM (*p* = 0.029) remained significantly associated with HM use in the 12 months preceding the interview. Adjusted odds ratios suggest potential associations, but the wide confidence intervals indicate uncertainty around the magnitude of effect.

## 4. Discussion

The current study assessed the prevalence, forms (presentations), and factors associated with HM use among adults with hypertension in an urban setting in Kampala, Uganda. In our study, the prevalence was 86(71.1%), representing the proportion of participants who had used HM at least once in the past 12 months, while the prevalence of exposure (defined as use at least once in a lifetime but not in the last 12 months) was 35(28.9%). This finding, although slightly below the 80% prevalence reported by the WHO for developing countries [18], aligns with other studies in Uganda that assess HM use in urban areas but not specifically for hypertension [34–36]. Comparisons with prevalence rates in rural settings indicate minor differences, such as Ref. [14], which reported a 56.2% prevalence of HM use among patients with hypertension in Buikwe, and Ref. [37], which reported 57.6% HM use among patients on ART therapy. Although other studies [12] note higher prevalence rates in rural settings overall, our findings highlight that HM use is not just a rural phenomenon but is deeply rooted in the modern urban healthcare landscape.

Residence in Central Uganda and ease of access to HM were significant factors associated with its use in this study. As the most commercially active region, Central Uganda hosts a large concentration of unregulated herbal vendors and traditional practitioners, a concern highlighted in the ND’s Guidelines for the Regulation of Local Traditional Herbal Medicines [22]. This may reflect the region’s pluralistic healthcare environment and the widespread access to both biomedical and traditional treatment options. In this context, accessibility seems to be enhanced by transportable forms, the proximity of gardens for herbs, and the involvement of family networks and friends. Importantly, access to herbalists, who often possess specialized knowledge of HM [13], underscores the need to re‐evaluate the pathways to access currently established in conventional health systems. References [38, 39] noted that treatment interventions for hypertension have been explored and shared across different health settings, including with some herbalists. It is not clear, however, what mechanisms exist to engage recognized herbalists to improve coordinated and safe care. Nonetheless, it is essential to understand how the interlinkages among patients’ context, knowledge of herbs, ease of access to herbs, access to herbalists, and support/influence from family and friends affect individual hypertension treatment choices. These factors, combined, indicate that decisions may not only depend solely on the patient and the conventional provider alone but also involve a combination of different sources of knowledge and influences.

Most participants in this study, 92 (76.0%), perceived HM as safe when used alongside conventional medicine, with 73 (79.4%) reporting use within the last 12 months (*p* = 0.001). This aligns with findings from other studies on a similar phenomenon [38, 40, 41]; however, despite widespread use and belief in the potential, safety, and perceived benefits of herbal remedies, Refs. [15, 42] emphasize that herbal treatments can be linked to serious adverse effects, sometimes leading to fatalities. Commonly reported adverse effects include gastrointestinal problems such as diarrhea and abdominal discomfort, skin conditions such as pruritus, urticaria, and rash, as well as more serious reactions such as organ toxicity, especially hepatobiliary toxicity, pulmonary issues, and allergic reactions [24, 25]. These reports highlight the importance of cautious use and monitoring when prescribing or consuming HMs, given potential safety concerns. Hence, more scientific evidence on risks, injuries, and adverse effects (including mortality) is vital to inform knowledge development and targeted health education initiatives. Healthcare professionals play an essential role in bridging the knowledge gap, especially about the benefits and potential disadvantages of using HM by patients [43]. The same authors stress that pharmacists, for example, can bridge the gap by sharing knowledge with specialists such as physicians and oncologists since they interface with HM users routinely.

Most participants in this study did not disclose HM practices to their providers or could not recall having such a conversation during a routine hypertension visit. The odds (AOR = 5.3; 95% CI = 0.5–54.0) of HM use were higher among those who did not notify or communicate with conventional health providers. This may reflect recall bias; however, consistent with other findings, many patients fail to disclose HM use to their healthcare providers due to the fear of disapproval or a lack of inquiry, thereby exacerbating the risks of adverse effects and compromised treatment outcomes [44]. Addressing this gap demands targeted interventions to improve provider–patient dialog about HM use, enabling safer integration with conventional therapies and informing tailored health education initiatives. To gain insights into practices and patterns related to HM use, conventional health workers need information, and efforts and opportunities should be made to establish rapport on this subject in routine interactions with patients.

In this study, participants’ use of HM increased even when they perceived it as having some risk or side effects. This may be explained by the fact that the majority of this cohort reported no side effects from repeated use of HM and may have prioritized benefits over risks. Perceived safety and the benefits of HMs have been documented elsewhere [14, 45, 46] as among the main reasons for using them. Rigorous evidence supporting the therapeutic benefits of HM, or its integration with conventional medicines, is urgently needed to close the gap between what patients report and the impact and outcomes of sustained HM use.

Additionally, both ease of access and perceived safety may be related to indigenous knowledge of both medicinal plants and traditional cures. For example, an ethnobotanical study in the Rwenzori region documented 77 medicinal plant species used by communities for over 67 diseases, many of which were widely accessible and familiar to the local population, often prepared simply by using leaves and boiling water [47]. Similarly, Ref. [48] highlighted a high dependency on a diverse range of medicinal plant species and traditional medicinal knowledge for healthcare and income generation. Critically, studies show that the growth of urban and peri‐urban populations in Uganda has increased the use of “food as medicine” as part of health‐seeking behavior, a trend similar to that observed in other sub‐Saharan African countries [49]. This phenomenon reflects a dynamic interaction between the use of nutritional and medicinal plants and a constant search for treatment options with widespread familiarity and open sharing of knowledge among communities. Thus, the growing and normalized reliance on food‐based medicinal plants may partly explain the high prevalence of HM use, the ease of access, disclosure practices, and perceived safety. However, it also creates a dual system where patients make important treatment decisions without their conventional provider’s knowledge. Future research should consider exploring these key connections within an integrated model of healthcare.

### 4.1. Strength of the Study

The strength of this study lies in its systematic assessment of HM use among patients with hypertension in an urban Ugandan setting, contrasting with previous studies that primarily focused on rural areas or other illnesses, thereby providing valuable insights for tailoring patient care and informing future research and targeted interventions.

### 4.2. Limitations of the Study

This study used a cross‐sectional design, which limits the ability to establish causal relationships and directionality of associations. Additionally, recall bias may have affected the findings, since factors related to HM use were assessed at the same time. Since the study focused on urban settings, the results may not apply to rural populations. However, other studies have examined HM use in rural areas, and this research aimed to add insights by focusing on less‐studied urban groups. Some of the effect estimates in this study had wide confidence intervals, indicating some degree of statistical imprecision. This may reflect the modest sample size or variability within the data. As such, these estimates should be interpreted with caution, and future studies with larger samples are needed to provide more precise effect estimates.

## 5. Conclusion

Hypertension patients in urban centers widely use HM, mainly because of perceptions of its benefits, safety, and ease of access. The perception that it is safe highlights the need for more research to systematically measure and track outcomes in patients using herbal remedies alone or alongside conventional treatments for hypertension. This has significant implications for healthcare policy and practice. Policymakers should consider integrating HM into national hypertension management strategies by establishing regulations, safety monitoring, and encouraging collaboration between herbalists and conventional healthcare providers. Clinicians should proactively inquire about and document HM use during patient visits to identify potential herb–drug interactions and customize treatments accordingly. This will support the development of safer, more effective integrated treatment approaches, ultimately improving hypertension management and patient safety.

## Disclosure

The content is solely the responsibility of the authors and does not necessarily represent the official views of the National Institutes of Health.

## Conflicts of Interest

The authors declare no conflicts of interest.

## Funding

Research capacity for John Bosco Alege, Florence Nakaggwa, and Rose Clarke Nanyonga for the research reported in this publication was supported by the Fogarty International Center of the National Institutes of Health, U.S. Department of State’s Office of the U.S. Global AIDS Coordinator and Health Diplomacy (S/GAC), and President’s Emergency Plan for AIDS Relief (PEPFAR) under Award Number 1R25TW011213. The content is solely the responsibility of the authors and does not necessarily represent the official views of the National Institutes of Health.

## Data Availability

To protect participants’ anonymity, data will be shared upon reasonable request. The contact person is Keneth Mugume: mugumekenneth@gmail.com.
